# Cross-validation of prediction equations for estimating the body mass index in adults without the use of body weight

**DOI:** 10.1371/journal.pone.0316610

**Published:** 2025-02-21

**Authors:** Júlio César Chaves Nunes Filho, Marilia Porto Oliveira Nunes, Robson Salviano de Matos, Daniel Vieira Pinto, Dyego Castelo Branco Holanda Gadelha Pereira, Thais Amanda Silva Pereira Castelo Branco, Geraldo Bezerra Da Silva Júnior, Janaina de Almeida Mota Ramalho, Elizabeth De Francesco Daher

**Affiliations:** 1 Medical Sciences Postgraduate Program, Federal University of Ceará, Fortaleza, Brazil; 2 Department of Clinical Medicine, Federal University of Ceará, Fortaleza, Ceará, Brazil; 3 Department of Nutrition, University of Fortaleza, Fortaleza, Ceará, Brazil; 4 Department of Biomedicine, Federal University of Ceará, Fortaleza, Ceará, Brazil; 5 Fortaleza City Hall, Education Department, Fortaleza, Ceará, Brazil; 6 Brazilian Hospital Services Company/ Federal University of Amazonas, Manaus, Amazonas, Brazil; 7 Medical Sciences and Public Health Graduate Programs, School of Medicine, University of Fortaleza, Fortaleza, Ceará, Brazil; University of Illinois Urbana-Champaign, UNITED STATES OF AMERICA

## Abstract

**Introduction:**

Body Mass Index (BMI) is a widely accepted measure by the World Health Organization for assessing body composition, as it provides critical insights into health risks, life expectancy, and quality of life. However, in resource-limited settings, access to weighing scales is often inadequate, and environmental conditions, such as unstable terrain, may hinder accurate weight measurements. In these contexts, alternative methods for estimating BMI become essential for effective health assessment. This study aimed to develop and validate equations to estimate BMI without relying on body weight, providing a practical tool for nutritional assessment where traditional methods are not feasible.

**Materials and methods:**

Adults aged 18 to 59 of both sexes were included. Variables like waist circumference, height, hip circumference, age, and weight were used for equation development and validation. Participants were divided by sex, with regression and validation subgroups for each. Statistical tests included Student’s t-tests, Pearson correlation, Stepwise Regression, Intraclass Correlation Coefficient, Weighted Kappa Coefficient, and Bland-Altman statistics.

**Results:**

The study included 810 adults, with 63% (576) women. No significant differences were found in paired comparisons between regression and validation subgroups for both sexes (p > 0.05). Four equations were proposed for BMI estimation: EM2 and EM3 for males, and EF2 and EF3 for females. All equations showed strong positive correlations (r > 0.90), significant at p < 0.05. Regression analysis revealed R^2^ values between 0.861 and 0.901 (p < 0.000). Intraclass Correlation Coefficient values indicated agreement of 0.961 and 0.972 (p < 0.05), with Weighted Kappa values showing substantial agreement of 0.658 and 0.711 for both sexes (p < 0.05).

**Conclusion:**

Adopting the proposed equations for estimating BMI in adults without using body weight is safe and effective for measuring this body measure in this population, particularly when weighing these individuals is not feasible.

## Introduction

There has been an increase in obesity in today’s society due to a preference for consuming excessive calories and a lack of physical activity [1]. These shifts indicate a move in epidemiology towards higher calorie consumption, seen in population changes and a simultaneous drop in malnutrition with a rise in obesity rates [2]. Central obesity is currently present in 41.5% of the global population. In the last thirty years, there has been a notable rise in central obesity, particularly within certain subcategories. These results offer pivotal insights for creating public health programs to lessen the effects of central obesity on overall population well-being [3].

Numerous research studies underscore the growing worldwide health and socioeconomic crisis caused by being overweight and obese, presenting a significant public health problem. Those with these conditions are at a greater risk of developing different diseases like high blood pressure, diabetes mellitus (DM), high cholesterol, and heart diseases [4,5].

The Body Mass Index (BMI) was introduced by the World Health Organization (WHO) in 1997 as a standard tool for assessing the nutritional status of populations [6]. Originally proposed by Belgian statistician Lambert Adolphe Jacques Quételet in the late 19th century, BMI remains widely used worldwide and is calculated by dividing weight (in kg) by height squared [7] There are several techniques available to determine nutritional status, such as skinfold measurements, bioelectrical impedance analysis (BIA), hydrostatic weighing (PH), gas plethysmography (PG), dual-energy X-ray absorptiometry (DXA), computed tomography (CT), and magnetic resonance imaging (MRI). Yet, applying these techniques in outpatient clinical settings is challenging due to expenses, limitations in equipment size, and possible body composition changes like surgery or edema [8].

Although BMI does not account for fat distribution in the body, it remains a commonly used measure of obesity due to its simplicity, requiring only weight and height measurements. Yet, each method offers unique and pivotal information about body composition, without providing a complete view [9].

Amid the current obesity and malnutrition crisis, scientists are working to enhance measures of body fat for earlier detection of obesity, focusing on simplicity and cost efficiency. Due to BMI’s crucial importance as the main tool for assessing anthropometric status, there is an urgent need for alternative methods to estimate BMI accurately without relying on body weight. Alternative methods for measuring BMI are essential in places without traditional scales, such as isolated communities or areas with challenging environmental conditions. A new method that calculates BMI without requiring body weight measurements or relying on subjective assessments could be groundbreaking. Furthermore, population databases containing anthropometric information can assist in estimating BMI in cases where weight measurements are not accessible.

BMI is widely used to assess body composition due to its simplicity and effectiveness. However, traditional BMI calculations, which rely on accurate weight measurements, face limitations in various scenarios. In remote areas or low-resource settings, access to calibrated scales is often inadequate, hindering valid and reliable assessments. For example, healthcare providers in rural clinics may lack basic equipment, forcing them to estimate weight visually, which compromises accuracy. Similarly, environments with unstable surfaces, such as disaster-affected regions, mobile healthcare units, or makeshift shelters, present challenges for using traditional scales. Furthermore, weighing patients can be impractical during home visits by healthcare professionals in areas with limited infrastructure or in cases involving patients with mobility difficulties, where obtaining accurate weight measurements is particularly challenging. Additionally, self-reported weights, commonly used in resource-limited environments, are often inaccurate, leading to potential errors in BMI calculations. These challenges highlight the need for an alternative method to estimate BMI without direct weight measurement, particularly in global health contexts where reliable tools are essential for assessing nutritional status.

Recent studies have associated arm circumference with BMI in children and adolescents, reporting significant findings when BMI was below 21 kg/m^2^ [10–12]. Additionally, research efforts have focused on validating BMI in adults using anthropometric equations, particularly in hospitalized patients to assess malnutrition [13–15]. Other studies have aimed to validate equations for estimating lean and fat mass, which, despite showing promise, have not yet been widely adopted in clinical practice [16–18]. This study aimed to validate a novel anthropometric equation designed to estimate BMI without relying on direct body weight measurements. The goal was to provide a practical alternative for assessing nutritional status in scenarios where scales are unavailable, such as resource-limited settings, or where weight measurements are unreliable, including mobile healthcare units or environments with unstable surfaces. By addressing these challenges, this study seeks to contribute to improved health assessments in diverse and often underserved populations.

## Methods

### Study type, location, period, and population

The research utilized a cross-sectional design conducted between December 2017 and December 2018, across four community sports centers and public squares.The research focused on approximately 4,000 adults, both male and female, aged between 18 and 59, who self-reported engaging in physical activity. An analysis determined that 359 adults would be required for the sample size. However, our total number of participants was larger, with 908 active adults, included in the study from 18/12/2017 to 28/12/2018.

### Design of the study

In the early stage of the study, the researchers reached out to the sports centers’ administration to provide a detailed explanation of the study’s goals and purpose. After obtaining approval from the relevant parties at the organizations, specific time frames for gathering data were set up, covering morning, afternoon, and evening shifts to ensure everyone had an equal chance to participate. Experienced researchers carried out data gathering on-site by approaching people randomly and guiding those who were interested in a designated and suitable area to guarantee privacy while collecting data. Attendees completed a partially structured survey providing sociodemographic information before undergoing body composition measurements. Each member of the research team received training to verify anthropometric data accurately, following the guidelines established by the latest standards [19].

To ensure the applicability of the results, we used specific inclusion and exclusion criteria in our study. Participants were between 18 and 65 years old, regularly attended gyms, were available during the data collection periods (morning, afternoon, or evening), and provided written informed consent to participate. Exclusion criteria also encompassed individuals with chronic diseases or acute conditions (n = 15) that could affect body composition, such as edema, recent use of diuretics, and pregnant women (n = 6) due to significant physiological changes during pregnancy that could influence anthropometric measurements. Individuals who could not complete the data collection protocol, due to unavailability or inability to follow the study’s instructions, were excluded (n = 29). Participants who had undergone bariatric surgeries (n = 14) or plastic surgeries (n = 34) were excluded from the study. Consequently, the final study sample consisted of 810 volunteers, representing both sexes.

### Data collection

We assessed height using a stationary wall-mounted stadiometer (Sanny® brand, Standard model), which extends up to 220 cm and has a precision of 1 mm. Participants stood barefoot with feet together, upright posture, and head neutral for accurate measurement [19]. For circumferential measurements, we used a non-extensible anthropometric tape (Sanny® SN-1040 model) with a precision of 0.1 cm. Waist circumference (WC) was measured around the narrowest part of the torso, above the navel and below the rib cage. Hip circumference (HC) was measured at the widest part of the hips, anatomically corresponding to the area around the greater trochanters of the femur, ensuring that the tape did not compress the skin [19]. Both measurements were taken twice, and if there was a discrepancy between the results, the average of the values was used. Using these values in centimeters, we also calculated the waist-to-height ratio (WHtR) by dividing WC by height and the waist-to-hip ratio (WHR) by dividing WC by HC. WHtR and WHR are considered indicators of health risks, such as heart disease, DM, and mortality, according to guidelines from the National Heart, Lung, and Blood Institute [20].

Body Mass was assessed using a digital scale, specifically the Tanita® Ironman BC 558 model. Volunteers were instructed to keep their feet bare, stand up straight, dress lightly, and remove any items that could impact the outcomes. BMI was calculated using the formula weight (in kilograms) divided by height squared (in square meters). BMI categories were determined based on World Health Organization criteria, indicating ranges for normal weight, overweight, and different levels of obesity [21].

### Development and cross-validation of prediction equations to estimate BMI

To create and confirm equations for predicting BMI without body weight, the sample was split into two categories according to sex: “male” and “female.” Each group was subdivided into “regression” and “validation” subgroups, allocating 80% and 20% of participants to each subgroup according to Pedhazur’s statistical model [22]. We developed the BMI estimation equations using regression groups for men and women and then validated them using validation groups. Initially, data similarity was confirmed by comparing the regression and validation groups. Subsequently, we performed correlation analyses to identify which variables had the most significant association with BMI, helping to choose anthropometric variables for the regression model. We used the stepwise regression model to find the variables, alone or together, that best explained or predicted BMI.

We developed BMI estimation equations using separate regression groups for men and women and subsequently validated these equations using independent validation groups. Initially, we assessed the comparability of the regression and validation groups by verifying the similarity of their data distributions. Following this, we conducted correlation analyses, including Pearson’s Correlation, to identify the anthropometric variables most significantly associated with BMI [23]. We then employed a stepwise regression approach to identify the combination of variables that best explained or predicted BMI.

We determined eligibility criteria based on equations that included a maximum of three anthropometric variables, aiming for the highest R^2^ values and lowest standard errors of the estimates (SEE). In regression analysis, R^2^, or the coefficient of determination, is a statistical measure that indicates how well the independent variables (in this case, the anthropometric measurements) explain the variability in the dependent variable (BMI). A higher R^2^ value means that the model explains a larger proportion of the variance in BMI, indicating a better fit for predicting BMI. We chose the BMI equation models and later used them in the validation groups to calculate the estimated BMI values for men and women. We compared the estimated mean BMI values with those calculated using the traditional BMI method of weight divided by height squared.

We performed correlation and linear regression tests to evaluate the connection between the estimation equations and the traditional BMI when there were no statistical variances between the average values.

Additional analyses included Intraclass Correlation Coefficient (ICC) tests to assess the reliability and reproducibility of the equations. Specifically, we employed a two-way mixed-effects model ICC with absolute agreement, aiming to validate a new BMI estimation method that could be replicated across different populations while preserving the absolute accuracy of BMI values. This model was chosen because it allows for the assessment of both variability among individuals and consistency between the traditional and estimated BMI measurements [24]. We also used weighted Kappa correlation coefficient tests to evaluate agreement on categorical variables and identify inconsistencies [25].

### Ethical considerations

Following the Helsinki Declaration, all participants followed guidelines for human research and gave written informed consent. The research was granted permission by the Ethics Committee at the Federal University of Ceará. It was a component of a broader study examining how body composition is distributed, and how hypertension affects the quality of life in physically active adults, identified by protocol number 78688117.0.0000.5054.

### Analysis of statistics

In this study model, we employed a 95% confidence interval, reflecting a significance level of 5% (P < 0.05). To determine the required sample size, the sample size calculation formula was applied: (n = N.Z^2^.p.(1 − p)/ Z^2^.p.(1 − p) + e^2^.N − 1), considering the total population (N), the event probability (p), the sampling error (e), and the z-value for the desired confidence interval. By substituting the values N = 4,000, a 95% confidence interval (Z = 1.96), a probability of 0.5 (for maximum variability), and a sampling error of 5% (0.05), the formula indicated that a sample of 351 adults would be sufficient for the study. The Kolmogorov-Smirnov and Levene tests were conducted to assess the normality and homogeneity of the data, respectively. To represent normally distributed data we used data from Means and standard deviations. We performed comparison tests of dependent samples using the paired t-test for repeated measures or the Wilcoxon test. We used the Pearson correlation test to assess the presence of linear associations between variables. The coefficients (r) were classified as follows: values of r from 0 to 0.10 indicated a negligible correlation, 0.11 to 0.39 indicated a weak correlation, 0.40 to 0.69 indicated a moderate correlation, 0.70 to 0.89 indicated a strong correlation, and values above 0.90 indicated a very strong correlation [23,26]. We employed simple linear regression to evaluate how well one variable can predict or clarify another. To evaluate the reliability of measurements, we employed the ICC, also referred to as the Reproducibility Coefficient (R). The CC allowed us to determine the percentage of overall variability attributed to the variable being measured. We classified the ICC values according to the following scale: < 0.50 indicates poor reliability, 0.50–0.75 moderate, 0.75–0.90 good, and > 0.90 excellent reliability [24]. We also performed a Bland-Altman analysis to assess agreement between measurement techniques. Furthermore, we performed the Weighted Kappa test, based on the categories defined by Landis and Koch, to evaluate the level of agreement between the categorical estimators. The Kappa coefficient was interpreted as follows: values less than 0 indicated poor agreement, values between 0 and 0.20 suggested slight agreement, values between 0.21 and 0.40 indicated fair agreement, values between 0.41 and 0.60 were considered moderate agreement, values between 0.61 and 0.80 denoted substantial agreement, and values between 0.81 and 1.00 indicated almost perfect agrément [25].

## Results

**Fig 1:**
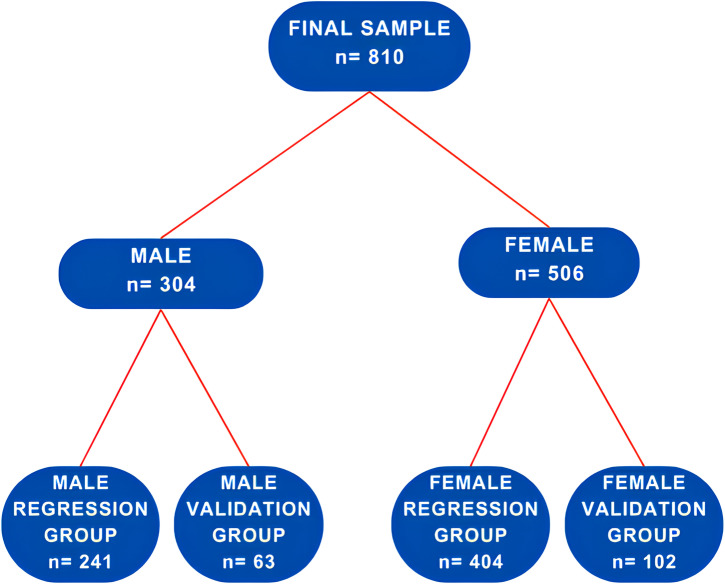
Displays a flowchart outlining how a group is divided for the development and validation of an equation to estimate BMI in adults.

We initially divided the 810 participants by sex into two groups: 304 males and 506 females, representing 63% of the total. These groups were further divided into regression and validation subgroups for developing and testing BMI prediction equations, as illustrated in Fig 1.

After categorizing the participants by sex, male (n = 304) and female (n = 506) participants were randomly assigned into two subgroups each: a ‘regression group’ and a ‘validation group.’ The details of these subgroups are shown in Table 1.

**Table 1:** Descriptive characteristics and comparison of variables between regression and validation groups: male and female.

Group	Regression		Validation		*p*
	m ± dp	med (q1–q3)	m ± dp	med (q1–q3)	
**Male**	**Validation (241)**		**Validation (63)**		
**Age (years)**	33.84 ± 9.6	33 (18**–**48)	33.56 ± 8.75	31 (27**–**29)	0.408
**Weight (kg)**	84.71 ± 13.48	83.1 (54.6**–**92)	84.67 ± 14.49	80.1 (76.1**–**90.6)	0.566
**Height (m)**	1.74 ± 0.06	1.7 (1.45**–**1.78)	1.74 ± 0.07	1.7 (1.69**–**1.79)	0.328
**BMI (kg/m**^**2**^)	27.88 ± 3.88	27.1 (19.8**–**30.22)	27.94 ± 4.08	26.6 (25**–**3.64)	0.319
**WC (cm)**	91.4 ± 10.24	91 (73**–**98)	90.76 ± 10.59	89.5 (83**–**98)	0.777
**HC (cm)**	102.57 ± 9.56	102 (98**–**107)	102.45 ± 9.09	101 (96**–**107)	0.488
**WHR**	0.88 ± 0.06	0.9 (0.76**–**0.92)	0.88 ± 0.07	0.9 (0.83**–**0.92)	0.281
**WHtR**	0.52 ± 0.06	0.5 (0.4**–**0.56)	0.52 ± 0.06	0.5 (0.47**–**0.56)	0.834
**Female**	**Regression (404)**		**Validation (102)**		
**Age (years)**	33.75 ± 8.3	33 (28**–**38)	34.23 ± 8.51	33 (28**–**40)	0.609
**Weight (kg)**	64.57 ± 10.99	62.25 (57**–**70.27)	66.44 ± 10.86	65.1 (59.25**–**71)	0.124
**Height (m)**	1.61 ± 0.06	1.61 (1.56**–**1.66)	1.61 ± 0.05	1.61 (1.58**–**1.65)	0.766
**BMI (kg/m**^**2**^)	24.74 ± 3.77	24.04 (22.21**–**26.72)	25.37 ± 3.55	24.75 (23.41**–**26.99)	0.126
**WC (cm)**	76.05 ± 9.24	74 (70**–**80.9)	77.51 ± 8.48	75.8 (71.75**–**81.62)	0.149
**HC (cm)**	101.25 ± 8.09	100.5 (96**–**106)	102.24 ± 7.29	101 (97**–**106)	0.262
**WHR**	0.75 ± 0.06	0.74 (0.70**–**0.77)	0.75 ± 0.05	0.74 (0.71**–**0.78)	0.364
**WHtR**	0.47 ± 0.05	0.46 (0.43**–**0.49)	0.48 ± 0.05	0.47 (0.44**–**0.51)	0.181

Legend: m, mean; dp, standard deviation; med, median; q1, first quartile; q3, third quartile; WC, waist circumference; HC, hip circumference; BMI, body mass index; WHtR, waist-to-height ratio; WHR, waist-to-hip ratio; p, significance value with 95% confidence interval, obtained by the independent samples t-test.

Furthermore, Table 1 presents the results of comparative evaluations of the means of the regression and validation subgroups for men and women, respectively.

We analyzed independent samples using Student’s t-test. For all variables studied in men and women — such as Age, Weight, Height, BMI, WC, WHR, and WHtR — comparisons did not show statistically significant differences (p > 0.05).

We found no significant differences (p > 0.05) in the means of the variables for men and women in the regression and validation groups, which supported the development of BMI estimation equations. We conducted correlation tests to examine the relationship between BMI and various anthropometric variables in both sexes within the regression groups. In men, BMI demonstrated a strong correlation with WC (r = 0.887, p < 0.001). For women, BMI exhibited a moderate yet significant correlation with WC (r = 0.649, p < 0.001). Age showed a weaker, yet significant, correlation with BMI (r = 0.218, p < 0.001), whereas height did not show a significant association with BMI (r = 0.036, p = 0.578).

In the female regression group, there were also strong correlations between BMI and WC (r = 0.847, p < 0.001) and HC (r = 0.793, p < 0.001). Age had a lower correlation (r = 0.271, p = 0.001), while height remained non-significant (r = −0.063, p = 0.206). We used Stepwise multiple regression analysis to identify which independent variables (age, WC, height, and HC) best predicted the dependent variable, estimated BMI. Table 2 presents the correlation coefficients (R), coefficients of determination (R^2^), and SEE for the eight equations, divided equally between the male and female regression groups. All equations presented strong correlation coefficients above 0.8, suggesting a significant correlation between the variables. In men, R values ranged from 0.887 to 0.912, with corresponding SEE values of 1.797 and 1.605. Similarly, in women, the models demonstrated R values ranging from 0.847 to 0.943 and SEE values ranging from 2.01 to 1.26.

**Table 2:** Equations for predicting BMI in different subgroups of males and females through regression analysis.

EQ	Stepwise Regression	R	R^2^	SEE
Male				
EM1	BMI = −2.923 + (0.338 * WC)	0.887	0.786	1.797
EM2	BMI = −2.844 + (0.358 * WC) + (−0.058 * AGE)	0.897	0.803	1.724
EM3	BMI = 11.274 + (0.371 * WC) + (−0.075 * AGE) + (−8,440 * HT)	0.906	0.819	1.653
EM4	BMI = 10.128 + (0.333 * WC) + (−0.060 * AGE) + (−9,477 * HT) + (0.058 * HC)	0.912	0.829	1.605
Female				
EF1	BMI = −1.576 + (0.346 * WC)	0.847	0.717	2.015
EF2	BMI = −11.843 + (0.234 * WC) + (0.186 * HC)	0.894	0.799	1.699
EF3	BMI = 12.574 + (0.233 * WC) + (0.227 * HC) + (−17.657 * HT)	0.942	0.887	1.277
EF4	BMI = 14.118 + (0.248 * WC) + (0.218 * HC) + (−18.263 * HT) + (−0.026 * AGE)	0.943	0.889	1.264

Legend: EM, equation for male sex; EF, equation for female sex; BMI, body mass index; WC, waist circumference; HT, height; HC, hip circumference; R, multiple correlation coefficient; R^2^, coefficient of determination; SEE, standard error of estimate.

Equations characterized by lower R values, higher SEE values, and those incorporating more than three predictor variables were excluded from validation, aligning with common practices in clinical settings. As a result, equations EM2 and EM3 were retained for male validation, while EF2 and EF3 were selected for female validation.

We validated the selected equations by applying them to the respective validation groups. We compared the model-derived BMI values with actual BMI values using the Student’s t-test for dependent samples. In the male validation group, we calculated a mean BMI of 28.01 ± 4.09 kg/m^2^. The comparison with BMI estimates from EM2 (27.65 ± 3.77 kg/m^2^) and EM3 (27.69 ± 3.85 kg/m^2^) showed no significant differences (P > 0.05). For female participants, we calculated a mean BMI of 25.37 ± 3.55 kg/m^2^, and comparisons with EF2 (25.41 ± 3.13 kg/m^2^) and EF3 (25.43 ± 3.29 kg/m^2^) also showed no significant differences (P > 0.05).

**Fig 2:**
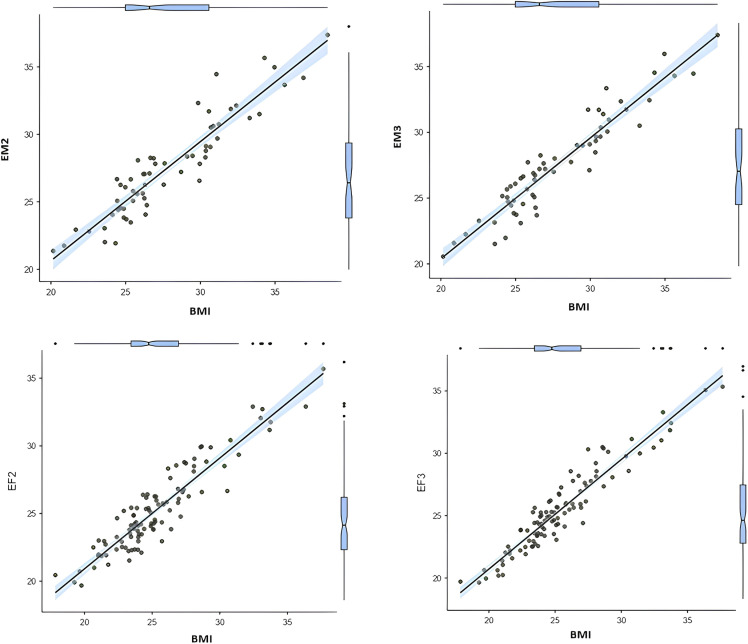
Correlation and prediction between the developed equation models and BMI in the validation groups of male and female. BMI, body mass index; EM2 and EM3, equation models 2 and 3 for estimating BMI in males; EF2 and EF3, equation models for estimating BMI in females.

Table 3 and Fig 2 present correlation values and predictions for the BMI equations EM2, EM3, EF2, and EF3. We observed very strong correlations (r > 0.9, p < 0.05) for all equations. Males showed a correlation of 0.929 for EM2 and 0.943 for EM3, while females showed a correlation of 0.928 for EF2 and 0.949 for EF3. Linear regression demonstrated strong predictive power, with R^2^ scores ranging from 0.863 to 0.890 for men and from 0.928 to 0.949 for women (p > 0.05). Furthermore, we observed low SEE values, ranging from 1.12 to 1.33 in females and from 1.36 to 1.52 in males.

**Table 3:** Correlation and regression between BMI estimation models in the validation subgroups of male and female sex and lambert Adolphe’s BMI.

Groups	Model	R	R^2^ adjusted	SEE	Change of R^2^	Change of F	*p*
Male validation	**EM2**	0.929	0.863; 0.861	1.52	0.863	377.912	0.0001
	**EM3**	0.943	0.890; 0.888	1.36	0.890	437.114	0.0001
Female validation	**EF2**	0.928	0.861; 0.859	1.33	0.861	617.04	0.0001
	**EF3**	0.949	0.901; 0.900	1.12	0.901	910.124	0.0001

Legend: r, pearson correlation coefficient; R^2^, coefficient of determination; p, significance value; SEE, standard error of estimate; F, joint statistics of independent variables added or removed from the regression model.

Further linear regression analysis highlighted the predictive accuracy of the models. In males, a comparison between EM2 and EM3 indicated an increase of 0.033 in R^2^, reflecting a statistically significant enhancement in BMI prediction with EM3 (p < 0.05). For females, EF3 provided a 0.044 increase in R^2^ over EF2, underscoring its superior predictive capability (Table 4).

**Table 4:** Comparison of regression models for BMI estimation in male and female sexes.

Groups	Comparison		ΔR^2^	F	gl1	gl2	p
	Model	Model					
Male	EM2	EM3	0.0335	19.5	1	58	< 0.001
Female	EF2	EF3	0.0447	46.8	1	99	< 0.001

Legend: ΔR^2^, the difference between the coefficients of determination; F, the statistical value of the model comparison; df1, degree of freedom associated with the model; df2, degree of freedom associated with the residual; P, significance value associated with the F test.

**Fig 3:**
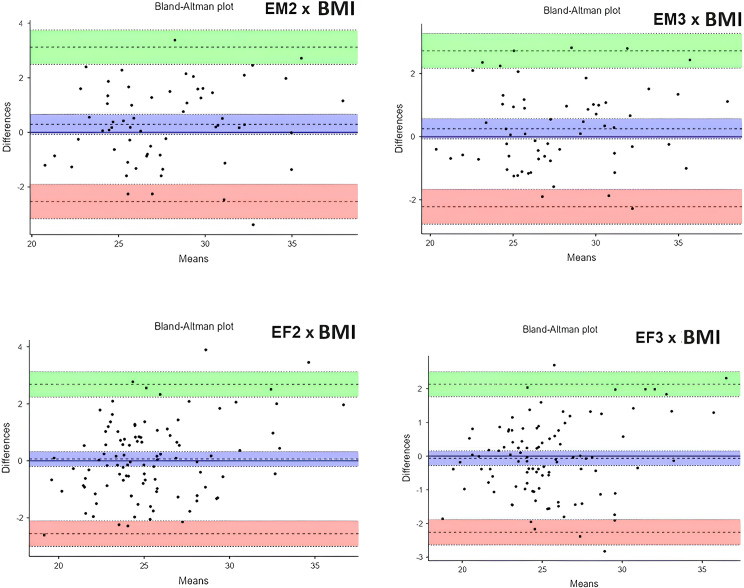
Scattering concordance between real and estimated BMI in male and female sex.

In Fig 3 we can observe the Bland-Altman analysis, used to evaluate the correlation between BMI estimation methods and conventional BMI in our study. Within the male validation group, the average discrepancy between estimated and actual BMI was 0.294 for EM2 and 0.251 for EM3. This suggests that on average, both models slightly overestimated BMI, but the discrepancies generally fell within acceptable limits. Within the female validation group, the mean difference between BMI predictions and actual BMI was 0.064 for EF2 and −0.063 for EF3, suggesting a slight disparity across both models. EF2 was usually slightly above the true BMI, while EF3 was usually slightly below the true BMI.

Table 5 presents the results of the ICC test comparing the proposed formulas for estimating BMI by sex. In this analysis, we aimed to evaluate the consistency between measurements obtained through methods in a scenario. In the validation group, we observed correlations, with p values < 0.05 with the strong agreement between the BMI and the estimated BMI. The mean ICC values were 0.926 (With a 95% CI: 0.880–0.955) for EM2 and 0.962 (With a 95% CI: 0.926–0.977) for EM3. Likewise, in the validation group, ICC analyses also showed robust results (p < 0.05) and findings with values of 0.960 (With a 95% CI: 0.922–0.955) for EF2 and 0.972 (With a 95% CI: 0.959–0.981), for EF3.

**Table 5:** Intraclass correlation coefficient between BMI and estimated BMI, for males and females.

Groups	Models		Intraclass correlation	Confidence interval 95%	f	p
Male Validation	**EM2**	M. Means	0.962	(0.936; 0.977)	26.032	0.001
	**EM3**	M. Means	0.970	(0.950; 0.982)	33.283	0.001
Female Validation	**EF2**	M. Means	0.960^c^	(0.933; 0.976)	24.804	0.001
	**EF3**	M. Means	0.972	(0.959; 0.981)	36.318	0.001

Legend: EM2, equation male validation group 2; EM3, equation male validation group 3; EF2, equation female validation group 2; EF3, equation female validation group 3; f, F-statistic used to verify group variability; CI95%, 95% confidence interval; p, significance value obtained by the intraclass correlation coefficient, with a 95% confidence interval.

Table 6 presents the results of the Weighted Kappa test, used to evaluate the level of agreement between BMI values and those predicted by the proposed anthropometric equations in the validation groups for both males and females. The test assesses the strength of agreement beyond chance, with values ranging from 0 (no agreement) to 1 (perfect agreement). All exams produced statistically significant results (p < 0.05). We found significant Kappa values in the female validation group, with EF2 achieving a score of 0.680 (95% CI: 0.557–0.803) and EF3 achieving a score of 0.711 (95% CI: 0.598–0.825), both indicating ‘substantial’ agreement. In the male validation group, EM2 and EM3 also presented noteworthy results (p < 0.05), with Kappa values of 0.680 (95% CI: 0.557–0.803) and 0.680 (95% CI: 0.529–0.787), respectively, both categorized as ‘substantial’ agreement.

**Table 6:** Weighted Kappa concordance evaluation between estimated BMI and traditional BMI by sex.

Groups	Models	Kappa	EP asymptotic	Z	IC95% LOW-UPP asymptotic	*p*
Male Validation	**EM2**	0.674	0.065	7.703	(0.547–0.801)	0.001
	**EM3**	0.658	0.066	7.478	(0.529–0.787)	0.001
Famale Validation	**EF2**	0.680	0.063	8.897	(0.557–0.803)	0.001
	**EF3**	0.711	0.058	9.484	(0.598–0.825)	0.001

Legend: EM2, equation validation group male 2; EM3, equation validation male 3; EF2, equation validation group female 2; EF3, equation validation female 3; IC95%, 95% confidence interval; p, significance value of the weighted kappa test; Z, Z-score of the Kappa test; EP, standard error.

## Discussion

Numerous studies have linked BMI to non-communicable chronic diseases, such as cardiovascular diseases, diabetes, hypertension, dyslipidemia, and chronic kidney disease. A recent study involving 62,957 adults found a significant positive correlation between BMI and hypertension [27]. Other research has also examined the association between BMI and conditions such as diabetes mellitus, dyslipidemia, and cardiovascular disease, identifying BMI as a significant risk factor for various health conditions [28–30]. Additionally, meta-analyses, such as those conducted by the Global BMI Mortality Collaboration with 10,625,411 participants and Flegal et al. with 2.88 million volunteers worldwide, have demonstrated a strong association between higher BMI and increased all-cause mortality rates [31,32].

BMI remains widely used for assessing disease risk and mortality in clinical medicine and epidemiology. Recent studies have also shown a correlation between higher BMI and increased mortality rates from COVID-19, further highlighting its relevance in managing patients infected with the virus [33–35].

Although widely used for assessing health risks, BMI has well-documented clinical limitations [36–38]. Due to its inability to differentiate between lean mass and fat mass, BMI can provide inaccurate classifications, particularly in populations with varying body compositions, such as those of different ethnicities, sexes, older adults, and muscular individuals, leading to either underestimation or overestimation of adiposity. In muscular individuals, BMI may overestimate adiposity, potentially misclassifying them as overweight or obese, despite having a healthy body composition. Conversely, in older adults or individuals with sarcopenia, BMI might underestimate adiposity, overlooking cases of excess fat mass and associated metabolic risks. These misclassifications can have significant implications for health assessments, potentially leading to inappropriate clinical decisions, such as unnecessary weight loss interventions or failure to address underlying metabolic conditions [36–38].

Over time, various models have been proposed to estimate body composition, mainly using anthropometric methods and equations designed to calculate fat and lean mass percentages [13–18]. Among these, studies with samples similar to ours have aimed to validate BMI in adults through anthropometric equations, focusing primarily on malnutrition in hospitalized patients [13–15]. On the other hand, other body composition validation studies have employed dual-energy X-ray absorptiometry (DEXA) as the reference standard, given its established role as a gold standard method for assessing body composition due to its high accuracy and reliability in differentiating between lean mass, fat mass, and bone mineral content [16–18]. However, despite its importance, DEXA-based models are often impractical in clinical settings. To address this gap, we validated an anthropometric equation based on critical factors such as sex, age, height, WC, and HC, following the NHLBI guidelines [20]. This approach allows for the calculation of BMI without the need for body weight, making it particularly useful in situations where traditional measurement methods are impractical.

Previous studies have indicated that body composition measurements can be influenced by physiological conditions, such as pregnancy, and pathological conditions, such as liver diseases and abdominal cosmetic procedures [39,40]. To minimize these biases in our validation research, participants were excluded with such conditions. To develop and validate the BMI estimation equation, we used the cross-validation model proposed by Pedhazur [22]. This model involves splitting the data into multiple subsets, training the model on some subsets, and testing it on others to evaluate its performance. A total of 810 volunteers participated in our research, a sample size comparable to other validation studies [33–40]. The participants were divided into male and female subgroups for validation and regression purposes. Independent sample comparisons revealed no statistically significant differences in anthropometric variables between the subgroups, supporting the application of the equations to both sexes. Correlation analyses within the regression subgroups showed significant correlations for WC, HC, and age in both sexes, with WC being the most strongly correlated variable.

Using the Stepwise Regression model, variables for the regression analysis and developed four predictive models for each sex were selected. In the female models, the inclusion of HC increased the accuracy of the predictions, possibly due to differences in visceral fat distribution between sexes, as previously described by other researchers [41]. We observed that the coefficients of determination (R^2^) for the proposed equations exceeded 85%, indicating high predictive capacity. This finding aligns with previous studies validating the use of BMI and body composition across various populations [33–41]. Furthermore, estimated standard errors (SEE), ranging from 1.12 to 1.52 for both sexes, indicates an accurate prediction according to Lohman’s criteria [42].

We excluded equations that presented less accurate predictions, higher estimated errors, and more than three anthropometric variables, following the inclusion criteria. This resulted in the validation models EM2 and EM3 for men, and EF2 and EF3 for women. Correlation and regression analyses revealed R coefficients between 0.929 and 0.943 for men, with R^2^ values ranging from 0.863 to 0.890, and R coefficients between 0.929 and 0.949 for women, with R^2^ values ranging from 0.861 to 0.901, indicating high reliability according to Koo and Li [26]. The more complex models (EF3 and EM3) demonstrated greater predictive accuracy compared to the simpler models (EF2 and EM2), although the simpler models also provided accurate predictions. Specifically, for males, the comparison between EM2 and EM3 revealed an increase in the coefficient of determination (R^2^) of 0.0335, with EM3 showing statistically significant improvement in BMI prediction (p < 0.001). Similarly, for females, EF3 showed a 0.0447 increase in R^2^ over EF2, further confirming its superior predictive capability (p < 0.001). Given clinical applications and data limitations, also decided to proceed with the more basic models.

Intraclass Correlation Coefficient (ICC) were used to assess the agreement between measurements in the development and validation of the BMI estimation equations. The ICC is widely recognized as a robust metric for assessing reproducibility and is considered excellent when its values exceeds 0.90 [43]. In the proposed equation models (EM2, EM3, EF2, and EF3), ICC values ranged from 0.96 to 0.97, indicating high consistency [44]. In the proposed equation models (EM2, EM3, EF2, and EF3), ICC values ranged from 0.96 to 0.97, demonstrating high consistency [44]. These results are consistent with other validation studies of anthropometric equations for estimating body composition, which reported ICCs ranging from 0.90 to 0.96 [39,40].

Finally, we used the weighted Kappa coefficient, following the method of Landis and Koch, to evaluate the agreement between the categorical qualitative classifications of the BMI estimation equations and traditional BMI [25]. In all the proposed equations, the Kappa values showed statistical significance.

Our study presents an innovative equation for estimating BMI without the need for body weight measurements. This method is particularly useful for vulnerable populations, such as those residing in remote rural areas and hard-to-reach locations, where resources and diagnostic equipment are limited. It complements previous research on anthropometric equations [13–15], providing a practical and accessible tool for monitoring nutritional status in these communities. Thus, it contributes to more accurate assessments and more effective interventions in environments with restricted access to diagnostic technologies.

Despite the promising results, our research has notable limitations. There was low representation of individuals classified as underweight (BMI < 18.5 kg/m^2^) and morbidly obese (BMI > 40 kg/m^2^), and did not include pregnant individuals or those with chronic or acute conditions presenting with edema. Although carefully segmented by sex to increase the accuracy of our equations, the inclusion of additional variables, such as ethnicity, could further enhance the validity of the proposed equations. In summary, while our goal was to estimate BMI without using body weight in adults, gaps remain regarding younger and older populations. We suggest that future research includes these age groups, such as older adults, children, and adolescents, as well as pregnant individuals and those with specific health conditions, to refine and broaden the application of the developed equations.

## Conclusions

To estimate BMI in adults, it is proposed four regression models, two for males and two for females, which were found to be relevant for application in healthcare contexts. Although these models have not been validated in traditional clinical settings, they are suitable for a diverse population, including individuals across different BMI categories (obese, overweight, underweight, and normal weight). These models provide a practical and accessible estimation of BMI, making them valuable tools for healthcare professionals in health and wellness monitoring programs.

To calculate BMI in males, EM2 included WC and age, while EM3 included WC, age, and height. In females, FM2 included WC and hip circumference (HC), while FM3 included WC, HC, and height. The selection of these variables was based on their strong correlations with BMI. WC emerged as the most significant predictor of BMI in both sexes, reflecting its role as a reliable indicator of central adiposity. In females, HC was also included due to its relevance in capturing the distribution of body fat, particularly around the hips, which is important in assessing BMI in women.

The proposed equations for estimating BMI in adults without the need for body weight measurement represent a safe and effective alternative, particularly in scenarios where direct weighing is unfeasible, such as among patients with mobility difficulties, individuals with significant physical limitations, or during fieldwork in resource-limited settings. The safety of these equations is inherent to their non-invasive methodology, which relies exclusively on anthropometric measurements, mitigating risks associated with patient handling or specialized equipment. Furthermore, their effectiveness is supported by their capacity to generate reliable BMI estimates that correspond closely with established reference methods, thereby facilitating the assessment of nutritional status and health monitoring across diverse contexts.

## Supporting information

S1 Text Female. (XLSX)

S2 Text Male. (XLSX)
